# Exploring Advance Directive Perspectives and Associations with Preferences for End-of-Life Life-Sustaining Treatments among Patients with Implantable Cardioverter-Defibrillators

**DOI:** 10.3390/ijerph17124257

**Published:** 2020-06-15

**Authors:** JinShil Kim, Hyung Wook Park, Minjeong An, Jae Lan Shim

**Affiliations:** 1 Department of Nursing, College of Nursing, Gachon University, 191 Hambakmoero, Yeonsugu, Incheon 21936, Korea; kimj503@gachon.ac.kr; 2 Department of Cardiovascular Medicine Chonnam National University Medical School, Chonnam National University Hospital, 42 Jebong-ro, Donggu, Gwangju 61469, Korea; mdhwp@jnu.ac.kr; 3 Department of Nursing, College of Nursing, Chonnam National University, Chonnam National University Hospital, 160 Baekseoro, Donggu, Gwangju 61469, Korea; 4 Department of Nursing, Dongguk University, 123 Dongdaero, Gyeongju-si, Gyeongsangbuk-do 38066, Korea

**Keywords:** implantable cardioverter-defibrillator, advance directive, advance care planning, perceived susceptibility, barriers/benefits

## Abstract

Deactivation of an implantable cardioverter-defibrillator (ICD) is a critical issue in the advance care planning (ACP) of ICD recipients; however, related perspectives have rarely been explored. Thus, this study aimed to provide an initial investigation of ICD recipients’ perceived susceptibility and barriers/benefits regarding ACP and/or advance directives (ADs), and associations of these modifiable factors with preferences for end-of-life life-sustaining treatments (LSTs) (cardiopulmonary resuscitation (CPR), ventilator support, hemodialysis, and hospice care). Using a descriptive correlational design, 48 ICD recipients (age, 50.1 years; male, 85.4%) completed survey questionnaires. “No burden on family” was the most highly valued (59.1%), followed by “comfortable death” (20.4%), and both (11.4%). LST preference was 43.8% for ventilator support, 45.8% for both hemodialysis and hospice care, and 54.2% for CPR. Perceived susceptibility to having unexpected end-of-life experiences increased the likelihood of preference for aggressive LSTs, with preferences increasing by 15% for CPR, 17% for ventilator support, and 23% for hemodialysis. A non-modifiable factor, older age, was the only predictor of increased preference for hospice care (odds ratio = 1.09, *p* = 0.016). Among the modifiable factors, a higher perceived susceptibility increased the likelihood of aggressive LST preferences. The findings imply that to facilitate informed decisions for LSTs, early ACP discussion could be helpful and enhance these modifiable factors.

## 1. Introduction

The survival benefit of an implantable cardioverter-defibrillator (ICD) has been proven, and it is widely used as a standard procedure today [1] for either primary or secondary prevention for sudden cardiac arrest [2,3,4]. Roughly 60,000 Americans have received ICD therapy [5], and 105,000 Europeans have received ICDs [6], either alone or in a hybrid form with biventricular pacing, which is increasingly being used for patients with advanced heart failure—particularly those undergoing ischemic cardiomyopathy who are at high risk of life-threatening arrhythmias [7]. The number of ICD implantations has increased over the past decade in South Korea too, with a total of 4649 ICDs implanted (31.2% for primary prevention and 68.8% for secondary prevention) [8]; however, overall, ICD therapy is underutilized in Asia [9]. ICD implantation remains underutilized as a primary prevention [10], though the rate of use has increased from 6.1% in 2007 to 41.9% in 2015 in South Korea [8].

However, a trade-off of the survival advantage of ICDs is often observed, which involves a broader spectrum of distressful post-implant adjustment associated with lifestyle changes as well as management of the underlying cardiac conditions and/or other comorbid conditions [11]. Furthermore, ICD recipients may face complex palliative medical decisions, including the deactivation of an ICD at the end-of-life (EoL) [12,13,14]. Particularly, the shock function of ICDs often occurs near death [15,16], which may lead to meaningless prolongation of life [12]. One approach to facilitate informed decision-making for future palliative care and avoid complicating EoL care is the early introduction of advance care planning (ACP) and/or advance directives (ADs) [12,14,17,18]. Through such a process, healthcare providers may better assist ICD patients and family members with ICD management [14,16] while also addressing the timely deactivation of ICDs as an essential component of ACP to improve patient care [11]. 

At the time of ICD implantation, discussions on EoL conditions could be initiated [12], which include, among other aspects, decisions about using an implant, the risks and benefits of this advanced therapy, and EoL care and device deactivation [17,19]. As part of ACP, such discussions can be undertaken during routine care when patients visit for periodical ICD interrogations and device management [18]. However, such discussions are rarely conducted until the impending death of recipients [12], often leading to situations where the device remains activated near EoL and deactivation is urgently requested [15,16]. A possible reason for the lack of attention to ACP/AD discussions is that ICD is primarily considered a life-saving therapy. Thus, its deactivation could be regarded as a complex and controversial issue, leading to delayed discussions and complicated EoL medical decisions [14]. The reported barriers to AD discussion from professionals, patients, and/or institutions are a result of lack of professional training, the knowledge deficit of patients regarding device deactivation [17], or a lack of a relevant policy or system guiding device deactivation [16]. Researchers have suggested accepting differences in opinions and/or attitudes to take into account individual perspectives and values [7]. Despite this, the perspectives of ICD recipients on ACP/AD and EoL care have rarely been considered in the literature. Therefore, the present study undertook an initial exploration of the ACP and/or AD perspectives of patients with ICDs. 

First, the present study aimed to examine the EoL values and life-sustaining treatment preferences (cardiopulmonary resuscitation (CPR), ventilator support, hemodialysis, and hospice care) of Korean ICD recipients; second, to compare differences in the levels of modifiable factors, perceived susceptibility, barriers, and benefits regarding ACP and/or ADs between patients who do and do not prefer these LSTs, and; third, to examine the associations with each LST, adjusting for significant demographic and clinical variables. 

## 2. Materials and Methods

### 2.1. Design and Procedure

A correlational study design was used to examine AD and/or ACP perspectives among patients with ICDs and the associations with preferences for EoL treatment options. Outpatients who received ICD therapy were invited to participate in this study when they visited a university-affiliated heart center for routine care and ICD interrogation/management. The institutional review board of the Chonnam National University Hospital approved this study protocol (CNUH-2016-293). Patients were asked to sign an informed consent statement prior to face-to-face interviews involving a survey questionnaire on ACP/AD and all subjects gave their informed consent for inclusion before they participated in the study. A trained research coordinator conducted interviews using a standard procedure decided by the investigators, which began with a brief introduction about the study, followed by oral administration of the survey questionnaire.

### 2.2. Subjects

The inclusion criteria for participation in the study was (1) aged ≥19 years, (2) post-ICD therapy of ≥3 months, and (3) ICDs in activation. Patients were excluded if one of the following criteria was met: (a) patients with an existing severe cognitive impairment associated with documented neuropsychological or cerebrovascular disorders which prevented active engagement in self-care, such as dementia/Alzheimer’s disease or brain or mental disorders; (b) recipients of biventricular pacing or a left-ventricular assisted device; (c) a heart transplantation candidate; or, (d) patients with a terminal comorbidity that qualifies for hospice care, such as terminal cancer, acquired immune deficiency syndrome, chronic obstructive pulmonary disease, or liver cirrhosis [20].

### 2.3. Measures

The Korean-Advance Directive (K-AD) model was used to explore EoL values and treatment wishes [21,22]. Patients were asked to consider their EoL value and indicate a preference for each of four treatment options—CPR, ventilator support, hemodialysis, and hospice care—on a dichotomous scale (1 = yes, 0 = no). 

The Advance Care Planning survey was used to measure perceived susceptibility, and perceived barriers and benefits were assessed using its subscales [23]. Perceived susceptibility refers to the extent to which a person perceives unexpected EoL experiences as important; it was assessed using the Perceived Susceptibility subscale (five items) [23]. Each item is measured on a seven-point Likert scale ranging from 1 (absolutely not important) to 7 (very important). Possible scores range from 5 to 35, with higher scores indicating a greater perceived importance for unexpected event experiences. The acceptable reliability (Cronbach’s alpha = 0.73) was determined based on reports from older adults [23].

Perceived barriers to and benefits of ACP/AD were assessed using the Perceived Barriers (nine items) and Benefits (seven items) subscales [24,25,26]. Each item is measured on a seven-point Likert scale ranging from 1 (very strongly disagree) to 7 (very strongly agree). Possible scores of the subscales range from 9 to 63 for barriers and 7 to 49 for benefits, with higher scores indicating greater perceived difficulties and usefulness of ACP and AD, respectively. The acceptable reliability was reported as Cronbach’s alpha coefficients of 0.91 for perceived barriers and 0.92 for perceived benefits [23,26].

Demographic information was obtained using a standard form developed by the investigators, which included age, sex, marital status, and educational level. For clinical information, ejection fraction, ICD implant date, ICD indication, and underlying cardiac illnesses were collected by reviewing electronic medical records. The Charlson Comorbidity Index was also employed to determine comorbidity scores [27]. The sum of weighted values for each condition was dichotomized, with multi-comorbid conditions of 0–1, and 2 and above. A trained research nurse also evaluated the functional limitations of patients using the New York Heart Association (NYHA) classification to classify the extent of limitations during daily activities from I (no limitation) to IV (severe limitation). To determine ICD recipient awareness, participants were asked whether they had prior knowledge or experience with ADs.

### 2.4. Statistical Analysis

A sample description was performed using descriptive statistics (i.e., frequencies, percentages, means, and standard deviations for normally distributed variables, and median and quartile 1 and 3 (Q_1_–_3_) for non-normally distributed variables). Content analysis was performed to determine the main themes of EoL values of ICD recipients, and their frequency and percentage were then calculated. The frequency and percentage of the EoL treatment wishes in the K-AD model were also calculated. Differences in the levels of perceived susceptibility and barriers to and benefits of EoL treatment choices (CPR, ventilator support, hemodialysis, and hospice care) were evaluated with Student *t*-tests. For predictive values of the AD-related variables of interest (perceived susceptibility, as well as barriers and benefits) for EoL treatment choices, multiple logistic regression analyses were performed with certain demographic and clinical characteristics (age, sex, ICD indication, post-ICD duration, and multi-comorbidity) and awareness of ADs as covariates. The Statistical Package for the Social Sciences Statistics for Windows, Version 25.0 (IBM Corp., Armonk, NY, USA) [28] was used to conduct all statistical analyses, with the level of statistical significance set at a *p*-value of 0.05. 

## 3. Results

Forty-eight patients with ICDs completed the survey questionnaires, including the K-AD model. The mean age of the ICD recipients was 50.1 years (±0.94). The majority of the participants were male (85.4%) and married (87.5%) (Table 1). The median left ventricular ejection fraction was 62.7% (Q_1–3_ = 47.2–67.4) and the median time after ICD implantation was 30.0 months (Q_1–3_ = 13.0–63.5). The indication of ICD therapy was 12.5% for primary prevention and 87.5% for secondary prevention of sudden cardiac arrest. Functional limitation measured by the New York Heart Association (NYHA) classes were none (83.3%, class I) and mild (16.7%, class II). Awareness of ADs was substantially poor, with only 20.8% of ICD recipients reporting that they had heard of ADs.

### 3.1. End-of-Life Values and Treatment Wishes

Korean patients with ICDs highly valued “no burden on family including children” (n = 26, 59.1%), followed by “comfortable death during EoL moment” (20.4%) and “dying comfortably without a burden to their family” (11.4%) (Table 2). Among the four treatment wishes, 54.2% of the ICD recipients reported a preference for CPR, 43.8% for ventilator support, 45.8% for hemodialysis, and 45.8% for hospice care. 

### 3.2. Differences in Perceived Susceptibility, and Barriers and Benefits According to Treatment Preferences

Perceived susceptibility and barriers were higher in participants with aggressive treatment preferences (Table 3). Specifically, perceived susceptibility significantly differed between those who did and did not prefer aggressive treatments, with the former showing higher scores. The level of perceived barriers was only significantly different between those with and without CPR and hemodialysis preference, with ICD recipients with CPR and hemodialysis preferences showing higher scores. However, no differences were found in perceived benefits between those with and without aggressive treatment preferences, and none of the modifiable factors significantly differed with hospice care preference. 

### 3.3. Predictive Values of Perceived Susceptibility and Barriers/Benefits for End-of-life Treatment Preferences

In a series of multiple logistic regression analyses (Table 4), after controlling for age, sex, comorbidity, ICD indication and duration, and AD awareness, perceived susceptibility was a significant predictor of CPR, ventilator support, and hemodialysis preference, while none of the modifiable factors significantly predicted a preference for hospice care. A one-score increase in perceived susceptibility was associated with a 15% increase in the odds of preferring CPR (odds ratio (OR) = 1.15, 95% confidence interval (CI) = 1.004–1.311), 17% increase in that of ventilator support (OR = 1.17, 95% CI = 1.025–1.337), and 23% increase in that of hemodialysis (OR = 1.23, 95% CI = 1.049–1.430). Among the non-modifiable factors, being female was significantly associated with less preference for hemodialysis (OR = 0.05, 95% CI = 0.003–0.897). Older age (OR = 1.09, 95% CI = 1.017–1.178) was associated with the likelihood of preferring hospice care, and the odds increased by 9% with a one-year increase in age. 

## 4. Discussion

The present study addressed EoL values, EoL preferences for LSTs, and several modifiable factors associated with LST preferences among ICD recipients in South Korea. Approximately half of the recipients showed a preference for aggressive treatments (CPR, ventilator support, and hemodialysis) while less than half preferred hospice care. Among the modifiable factors, perceived susceptibility was associated with an increased preference for CPR and hemodialysis, while a non-modifiable factor, older age, was the only predictor of increased preference for hospice care. Thus, our findings suggest that early introduction to an AD at implantation could help facilitate informed decisions for LSTs.

Device deactivation is a critical element of ACP discussions in ICD recipients [11,14,16], but related evidence was sparse in both Western and Asian countries. This lack of discussions often leads to recipient devices being active near EoL, inducing urgent requests for deactivation [15,16]. Limited care at EoL is more likely for those who indicate their preferred EoL care on an AD than for those without ADs [29]. Thus, we explored ACP and/or AD perspectives among ICD recipients and the influential factors for preferred LSTs at EoL. Foremost, poor understanding of ADs was substantiated in this study, with only one in five ICD recipients reporting their awareness of ADs. In South Korea, attention to ADs has increased since the recent enforcement of the Act on Decisions on Life-Sustaining Treatment Life-Sustaining Treatment Determination [20]. However, ICD recipients still demonstrated poor awareness, possibly because ICDs are perceived as lifesaving devices whereas ADs are perceived as inaccessible. One strategy for increasing awareness is to initiate ACP discussions and to integrate such discussions into routine care [18]. 

Regarding EoL values, more than half of the ICD recipients in this study highly ranked “no burden on family,” followed by “comfortable dying” (20.4%) and both (11.4%). Previous studies reported similar EoL values for cancer patients, but subtle differences were noted in comfortable dying (73.8%), which was primarily valued the most, and pain relief, which was primarily desired the most (47.4%), followed by no burden on family (21.1%) and both (5.3%) [22]. Community-dwelling chronically ill elders [22] and patients with heart failure [30] also highly valued comfortable dying (35.0% and 48.4%, respectively), followed by no burden on family (28.6% and 19.4%, respectively). Furthermore, approximately half of the respondents preferred aggressive treatments such as CPR, ventilator support, and hemodialysis. The ICD recipients in this study were more favorable to aggressive treatments (43.8–54.2%) compared to those in previous ones on patients with solid and hematologic cancer, chronically ill older adults, and patients with heart failure [22,31,32]. Compared to ICD recipients, aggressive treatments were less likely to be chosen among populations with both non-malignancy and malignancy. For example, in previous research, preferences for CPR and ventilator support equaled 41.7% and 27.8%, respectively, for outpatients with heart failure [31], followed by 20.5% for both treatments in outpatients with cancer [22] and only 6.8% and 4.5% in hospitalized patients with hematologic cancer, respectively [32]. Particularly, CPR preference in this study was much more prevalent among the ICD population (54.2%) compared to previous reports, with 41.7% of patients with heart failure [31] and 6.8% of patients with hematologic malignancy indicating such preference [32]. However, hospice care preference was relatively low among the ICD recipients (45.8%) compared to patients with heart failure (69.4%) [31] and outpatients with cancer (79.5%) [22]. These results from the current and prior studies indicate that EoL treatment preferences vary by diagnostic context and imply that informed decision-making for future EoL care should be encouraged through early ACP discussions considering the diagnostic context in both clinical and community settings. 

Some known modifiable and non-modifiable factors that could influence decisions about ACP/ADs or specific EoL treatments were preliminarily examined in patients with ICDs. Among the modifiable factors, perceived susceptibility was a significant predictor of preference for all aggressive treatments, including CPR, ventilator support, and hemodialysis, while no modifiable factors predicted a preference for hospice care. An increased perceived importance among ICD recipients of experiencing unexpected events at EoL increased the likelihood of aggressive treatments. Among the non-modifiable factors of demographics and ICD-related factors, being male increased the odds of preference for hemodialysis, and an older age was associated with increased preference for hospice care; meanwhile, none of the ICD-related factors were associated with preference for LSTs. Previous patient data on these factors is lacking, particularly with the use of reliable and valid measures in non-malignancy contexts [33,34,35,36]. In this context, this study identified perceived susceptibility as a significant predictor for considering ADs. Although we could not confirm the predictive values of other modifiable factors for LST preferences, an EoL discussion was more likely to occur among older adults with a high level of perceived barriers [23], while a desire for AD completion was less likely, with more perceived barriers among patients with hematologic disorders [37]. More empirical evidence for the influence of these modifiable factors on EoL care, including specific decisions about LSTs, is needed to design interventions for ACP discussion and AD completion in various populations. 

Few non-modifiable demographic factors, including older age and being female, were reported as significant when having ACP and/or AD were more likely. Consistent with this study, a prior study examined factors related to reduced time for hospice enrollment among older ICD patients and found older age to be an important factor, with other factors including advanced heart failure (NYHA class IV) and poor cardiac function (ejection fraction <20) [13]. Although females showed a reduced preference for hemodialysis in the present study, older age and being female were associated with more AD documentation among patients with heart failure, and so were being unmarried, being white, having a higher socioeconomic status [38], and having or intending to have ADs [39]. For those who possessed ADs receiving aggressive care (i.e., ventilator support or intensive unit care), near death was less likely [40]. Having palliative consultation also increased comfort care choices (i.e., hospice care or *do-not-resuscitate* order) [41]. Thus, offering ACP and/or ADs to ICD patients will help identify their palliative care needs throughout the course of their illness [13]. This study filled the knowledge gap by identifying perceived susceptibility, age, and sex as associated with preferences for EoL LSTs among ICD recipients that should be considered during ACP and AD-related care planning and education. Our results support the need to address ACP and ADs as early as possible and show that periodic discussions are more likely to enhance informed decision-making for future EoL care. However, our results were derived from a sample that contained relatively younger recipients and more women than the sample used in a previous study that used data from the National Health Insurance Service database [42]. Due to the influences of age and sex on the ACP discussion or AD utilization [38,39], the validation of our study results is warranted in a representative sample. 

### 4.1. Implications for Research and Practice

Since the amendment of the Act on Hospice and LSTs [20], awareness of advance planning for LSTs for the general population, and LST directives for the terminally ill, has increased. Our study provides additional insights into the ACP and ADs among Korean patients with ICDs and has significant implications for research and practice as it demonstrates the importance of addressing these issues among ICD patients. To facilitate informed decisions on LSTs and their eventual deactivation, introduction to an AD at implantation should be emphasized to address patients’ device-related concerns regarding future EoL care; simultaneously, the modifiable factors must also be enhanced. Due to a relatively high preference for aggressive treatments—particularly CPR—which may be attributed to the defibrillator being a life-saving therapy, in-depth, qualitative exploration about ACP and/or ADs among recipients and their families is needed. ACP models for ICD recipients should be developed to better assist recipients in living with ICDs and preparing for future EoL care. Efforts for legal support and to develop institutional policies to provide ACP and ADs for ICD recipients can begin with discussions by healthcare professionals regarding the full range of implications of ICD therapy, including device deactivation at the end of life.

### 4.2. Limitations

The present study used a small sample of patients recruited from a single hospital, which limits the generalizability of our findings to other populations. Thus, our findings should be verified with a larger representative sample. Furthermore, most patients used ICDs for secondary prevention, and only 12.5% of patients used ICDs for primary prevention which is indicated for those with advanced heart failure and a high risk of cardiac arrest. Perspectives for ACP and ADs could differ by ICD indication, indicating the need for a comparative investigation between the two groups of primary and secondary ICD recipients. 

## 5. Conclusions

ICD recipients in the present study preferred aggressive treatments. More than half preferred CPR the most and less than half preferred hospice care the least. Among the modifiable factors, perceived susceptibility to AD use increased the likelihood of preference for aggressive LSTs including CPR, ventilator support, and hemodialysis; among non-modifiable factors, being male was the only significant predictor of hemodialysis preference. Meanwhile, none of the modifiable factors were associated with hospice care preference for which the only predictor was older age. Our study initially examined ICD recipients’ EoL values and LST preferences and modifiable/non-modifiable factors that could affect decisions regarding LSTs. The findings provide initial insights into perspectives regarding ACP and ADs among Korean ICD recipients.

## Figures and Tables

**Table 1:** Demographic and clinical characteristics of the implantable cardioverter-defibrillator recipients (*N* = 48).

Variables		n (%)	Mean ± SD	Range
Age, years			50.1 ± 0.94	29.0–73.0
Sex	Male	41 (85.4)		
Marital status	Married	42 (87.5)		
Education	≤Middle school	6 (12.5)		
	High school	23 (47.9)		
	≥College	17 (35.4)		
NYHA classes	I	40 (83.3)		
	II	8 (16.7)		
	III/IV	0 (0.0)		
ICD indication	Primary	6 (12.5)		
	Secondary	42 (87.5)		
Underlying cardiac diseases	Ventricular arrhythmias	39 (81.3)		
	Brugada syndrome	3 (6.2)		
	Non-ICM	3 (6.2)		
	ICM	2 (4.2)		
	DCM	1 (2.1)		
Multicomorbidity	0–1	37 (77.1)		
	≧2	11 (22.9)		
ADs awareness	Yes	10 (20.8)		
		Median (Q_1_–Q_3_)		
LVEF (%)		62.7 (47.2–67.4)		
Post-ICD duration (months)		30.0 (13.0–63.5)		

Abbreviation: DCM, dilated cardiomyopathy; ICM, ischemic cardiomyopathy; LVEF, left ventricular ejection fraction; NYHA, New York Heart Association; SD, standard deviation. Q1, 1st quartile; Q3, 3rd quartile.

**Table 2:** End-of-life values and treatment preferences of the implantable cardioverter-defibrillator recipients.

Value statements (*N* = 44)		*n* (%)
No burden on family including children		26 (59.1)
Comfortable death during end of life		9 (20.4)
Dying comfortably without burden on family		5 (11.4)
Being buried after death		2 (4.5)
Never thought about it		1 (2.3)
Having a quiet funeral		1 (2.3)
Treatment wishes †	Cardiopulmonary resuscitation (Yes)	Treatment wishes †
(*N* = 48)	Ventilator support (Yes)	(*N* = 48)
	Hemodialysis (Yes)	
	Hospice care (Yes)	

† Multiple responses.

**Table 3:** Differences in perceived susceptibility and barriers/benefits according to end-of-life treatment preferences.

Variables	CPR Mean (±SD)		*t (p)*	Ventilator Support Mean (±SD)		*t (p)*	Hemodialysis Mean (±SD)		*t (p)*	Hospice Mean (±SD)		*t (p)*
	Yes (*n* = 26)	No (*n* = 22)		Yes (*n* = 21)	No (*n* = 27)		Yes (*n* = 22)	No (*n* = 26)		Yes (*n* = 22)	No (*n* = 26)	
Perceived susceptibility	24.46 (±6.62)	19.59 (±7.18)	2.44 (0.019)	25.19 (±6.98)	19.92 (±6.68)	2.66 (0.011)	25.27 (±6.74)	19.65 (±6.73)	2.88 (0.006)	23.95 (±7.36)	21.61 (±7.22)	0.64 (0.529)
Perceived Barriers	35.65 (±11.61)	27.54 (±13.70)	2.22 (0.031)	35.28 (±12.77)	29.33 (±13.03)	1.58 (0.120)	36.59 (±11.00)	28.00 (±13.68)	2.37 (0.022)	33.04 (±10.89)	31.00 (±14.92)	0.53 (0.596)
Perceived Benefits	34.00 (±7.37)	31.09 (±10.06)	1.15 (0.255)	34.23 (±7.34)	31.44 (±9.64)	1.10 (0.276)	33.9 (±7.7)	31.65 (±9.56)	0.87 (0.388)	33.27 (±8.03)	32.15 (±9.42)	0.44 (0.663)

Abbreviation: CPR, Cardiopulmonary resuscitation; SD, standard deviation; ADs, advance directives.

**Table 4:** Associations of perceived susceptibility and barriers/benefits with end-of-life treatment preferences (N = 48).

Outcome Variable	Factor	B	*p*	OR	95% CI	
					Lower	Upper
Cardiopulmonary	Constants	−4.83	0.132	0.01		
resuscitation	Perceived susceptibility	0.14	0.043	1.15	1.004	1.311
	Hosmer and Lemeshow’s Goodness-of-Fit test: χ^2^ = 3.58, *p* = 0.893; Nagelkerke *R*^2^ = 0.415					
Ventilator	Constants	−2.78	0.343	0.06		
support	Perceived susceptibility	0.16	0.020	1.17	1.025	1.337
	Hosmer and Lemeshow’s Goodness-of-Fit test: χ^2^ = 14.94, *p* = 0.060; Nagelkerke *R*^2^ = 0.320					
Hemodialysis	Constants	−3.06	0.361	0.05		
	Sex (female)	−2.92	0.042	0.05	0.003	0.897
	Perceived susceptibility	0.20	0.010	1.23	1.049	1.430
	Hosmer and Lemeshow’s Goodness-of-Fit test: χ^2^ = 8.42, *p* = 0.394; Nagelkerke *R*^2^ = 0.485					
Hospice care	Constants	−2.32	0.432	0.10		
	Age	0.09	0.016	1.09	1.017	1.178
	Hosmer and Lemeshow’s Goodness-of-Fit test: χ^2^ = 6.74, *p* = 0.565; Nagelkerke *R*^2^ = 0.383					

Abbreviations: CI, confidence interval; OR, odds ratio; ICD, Implantable Cardioverter Defibrillator; AD, advance directives. Note. Each model includes age, sex, ICD indication, ICD duration, multicomorbidity, AD awareness, perceived susceptibility, perceived barrier, and perceived benefit as predictive variables. References: Sex = male, ICD indication = primary prevention, Multicomorbidity = 0–1, ADs awareness = No.
